# Clinically induced hypothermia with cardiopulmonary support in a high-risk patient undergoing carotid endarterectomy

**DOI:** 10.1016/j.jvscit.2022.02.002

**Published:** 2022-03-04

**Authors:** Kjersti Hervik, Torvind Olav Næsheim, Truls Myrmel, Thomas Dammann, Ramez Bahar

**Affiliations:** a Department of Cardiothoracic and Vascular Surgery, University Hospital of North Norway, Tromsø, Norway; b Department of Anaesthesia, University Hospital of North Norway, Tromsø, Norway; c Medical Technical Department, University Hospital of North Norway, Tromsø, Norway

**Keywords:** Cardiopulmonary bypass, Carotid endarterectomy, Carotid stenosis, Induced hypothermia, Ischemic stroke

## Abstract

Contralateral carotid occlusion increases the risk of stroke by hypoperfusion in patients undergoing carotid surgery. We present the case of a high-risk patient with crescendo cerebral ischemic events, for whom clinically induced hypothermia controlled by cardiopulmonary bypass was applied as a protective measure during carotid endarterectomy.

Carotid stenosis is a major cause of cerebrovascular events, present in 20% of patients with ischemic stroke.^1^ Embolism is the main mechanism and the target of prevention when prophylactic treatment is performed by carotid endarterectomy (CEA). In the case of total occlusion, no embolization can occur, and CEA is not recommended. Nevertheless, a risk of watershed infarction caused by hypoperfusion exists. This occurs when the circulation cannot meet the brain’s oxygen demand, usually during episodes of hemodynamic alteration (ie, hypotension). Preventive treatment with CEA of the external carotid artery (ECA) or contralateral internal carotid artery (ICA) has previously been suggested^2^^,^^3^; however, the evidence is scarce.

In chronic ICA occlusion, the blood supply to the ipsilateral side of the brain will depend on the remaining precerebral arteries and collateral vessels from the ECA. Intracranially, the communicating arteries of the circle of Willis (CoW) feed into the anterior and middle cerebral arteries to maintain perfusion (Fig 1). Variations are common, and a complete CoW was found in only 11.9% of individuals in a recent magnetic resonance imaging study.^4^ If the ICA is clamped during CEA and contralateral occlusion is present, brain oxygenation can become compromised if the posterior contribution is insufficient. A shunt can be applied to maintain perfusion; however, its routine use has been associated with an increased stroke risk in some studies.^5^

**Fig 1:**
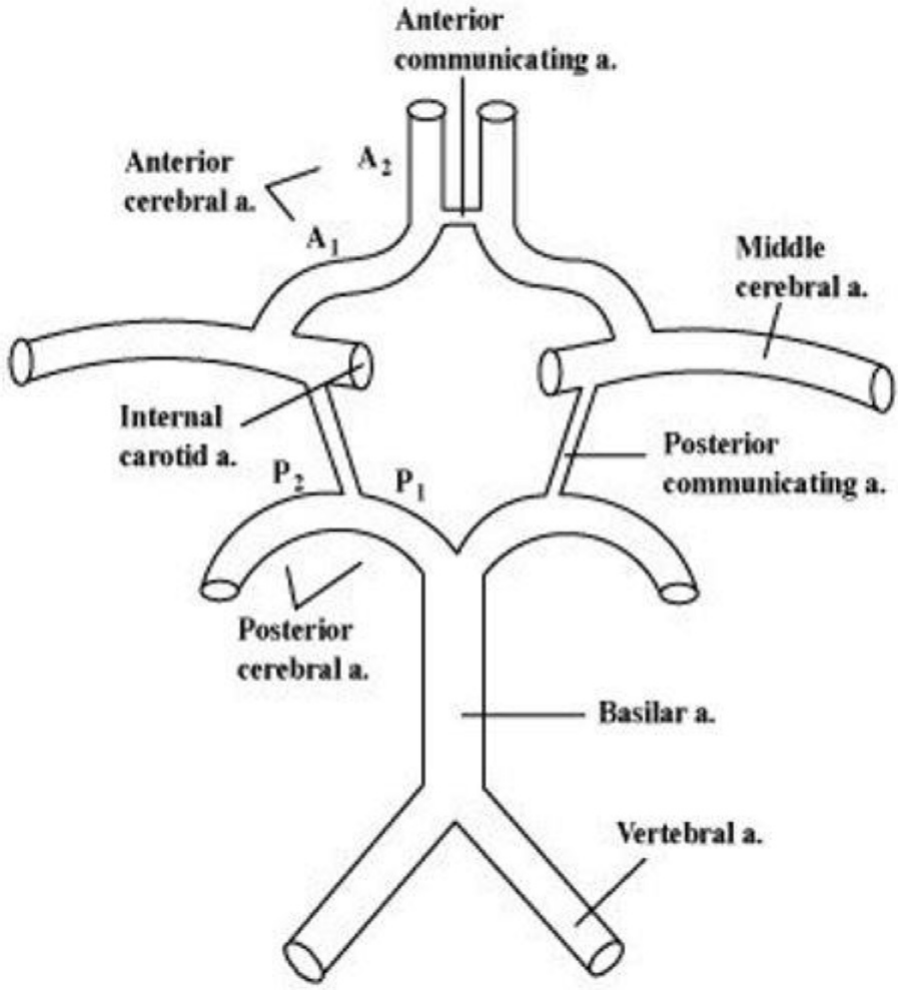
Complete circle of Willis (CoW), with intact communicating arteries between the right and left and anterior (*A*) and posterior (*P*) circulation. *a.,* Artery.

Controlled systemic hypothermia for end-organ protection is a well-established technique, such as during aortic arch surgery. Animal studies have previously shown reduced brain oxidative stress when regulated hypothermia is applied during hypoxic ischemia.^6^ It seems reasonable to assume the same protective effect would be present during carotid clamping. Moderate hypothermia has been suggested to be feasible and safe during CEA in a small previous study.^7^

We present the case of a patient with a high risk of hypoperfusion. The patient provided written informed consent for the report of her case details and imaging studies.

## Case report

A 62-year-old woman had been admitted to a community hospital in North Norway with acute weakness of her left arm and reduced right side vision. She had a history of depression but no somatic medical record. She was a current smoker. During the previous month, she had experienced several episodes of neurologic symptoms of short duration, such as left arm weakness, a reduced visual field, and numbness of her face and left arm. She woke up with a lasting sensation of a shadow in her right visual field and consulted a doctor the following day. On clinical examination, her blood pressure was 205/103 mm Hg, and she had slightly reduced power in her left arm and impaired sensation of her left face, upper arm, and calf. She had left-sided neglect on the finger–nose test a bilateral deficit in visual fields and was unable to focus and follow on testing of eye movement. The electrocardiogram showed sinus rhythm. She had hypercholesterolemia, with total cholesterol of 8.2 mmol/L and low-density lipoprotein cholesterol of 6.3 mmol/L.

**Fig 2:**
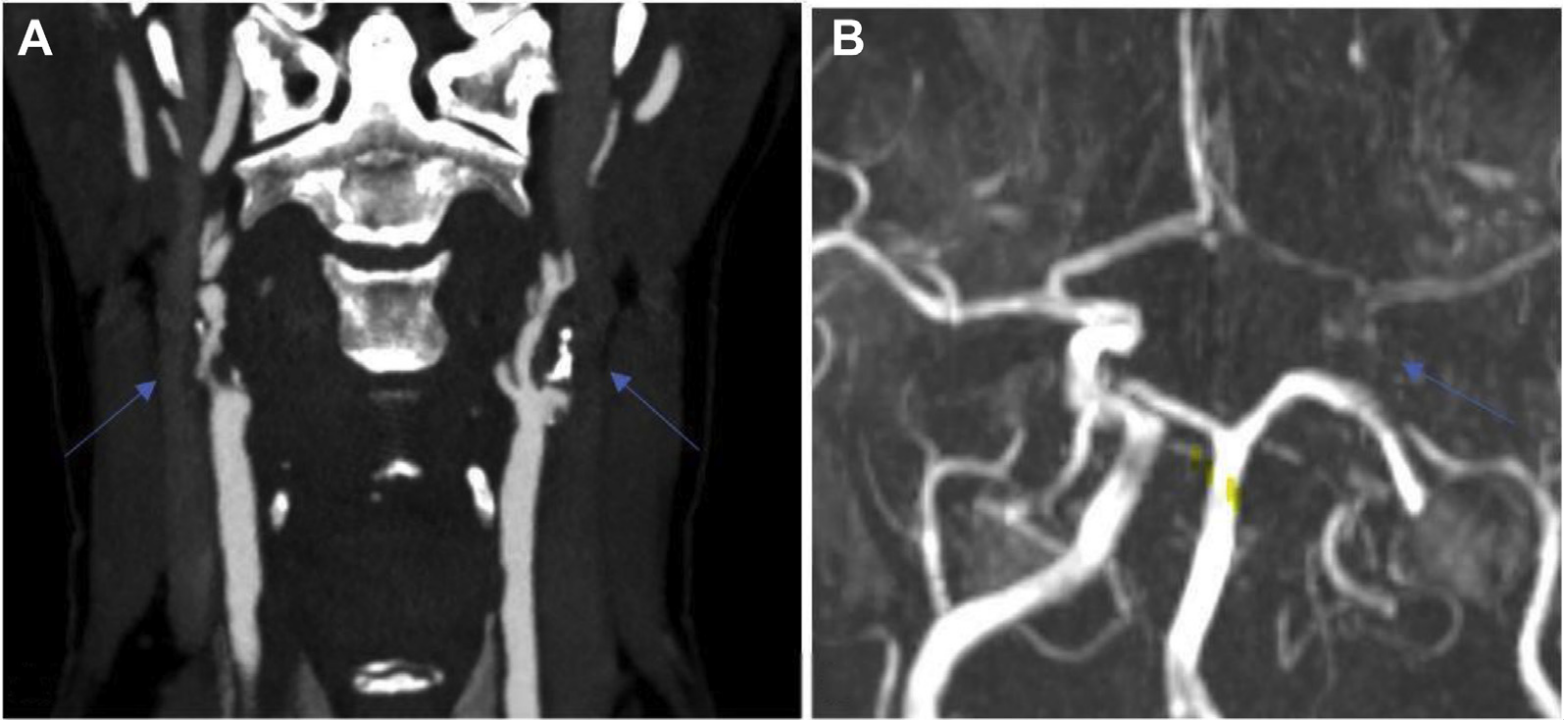
**A,** Computed tomography angiogram of the neck showing a calcific occlusive plaque in the proximal left internal carotid artery (ICA) and near occlusion of the proximal right ICA (*arrows*). **B,** Magnetic resonance imaging study showing an incomplete circle of Willis (CoW; *arrows*).

Magnetic resonance imaging revealed a new area of infarction in the right cortical watershed area and the area supplied by the middle cerebral artery. Also, several small areas of frontoparietal infarction were present. Computed tomography angiography showed occlusion of the left ICA and right vertebral artery and 90% right ICA stenosis, with a partially calcified plaque (Fig 2, *A*). Incomplete contrast filling of the CoW’s left posterior communicating artery was present (Fig 2, *B*).

The risk of recurrent strokes by embolization from the right ICA or global hypoperfusion was considered significant. However, the risk of ischemia during carotid artery clamping was concerning, and the small caliber of the distal right ICA made it uncertain whether a shunt could be safely applied.

Clinically induced hypothermia was suggested for cerebral protection. Further investigation by coronary angiography showed moderate stenoses of the left main, circumflex and right coronary arteries. Cannulation for cardiopulmonary bypass (CPB) was planned to induce and control hypothermia and to meet the risk of circulatory collapse if ventricular fibrillation should occur. The patient was informed of the treatment decisions and provided consent to the choice of management.

**Fig 3:**
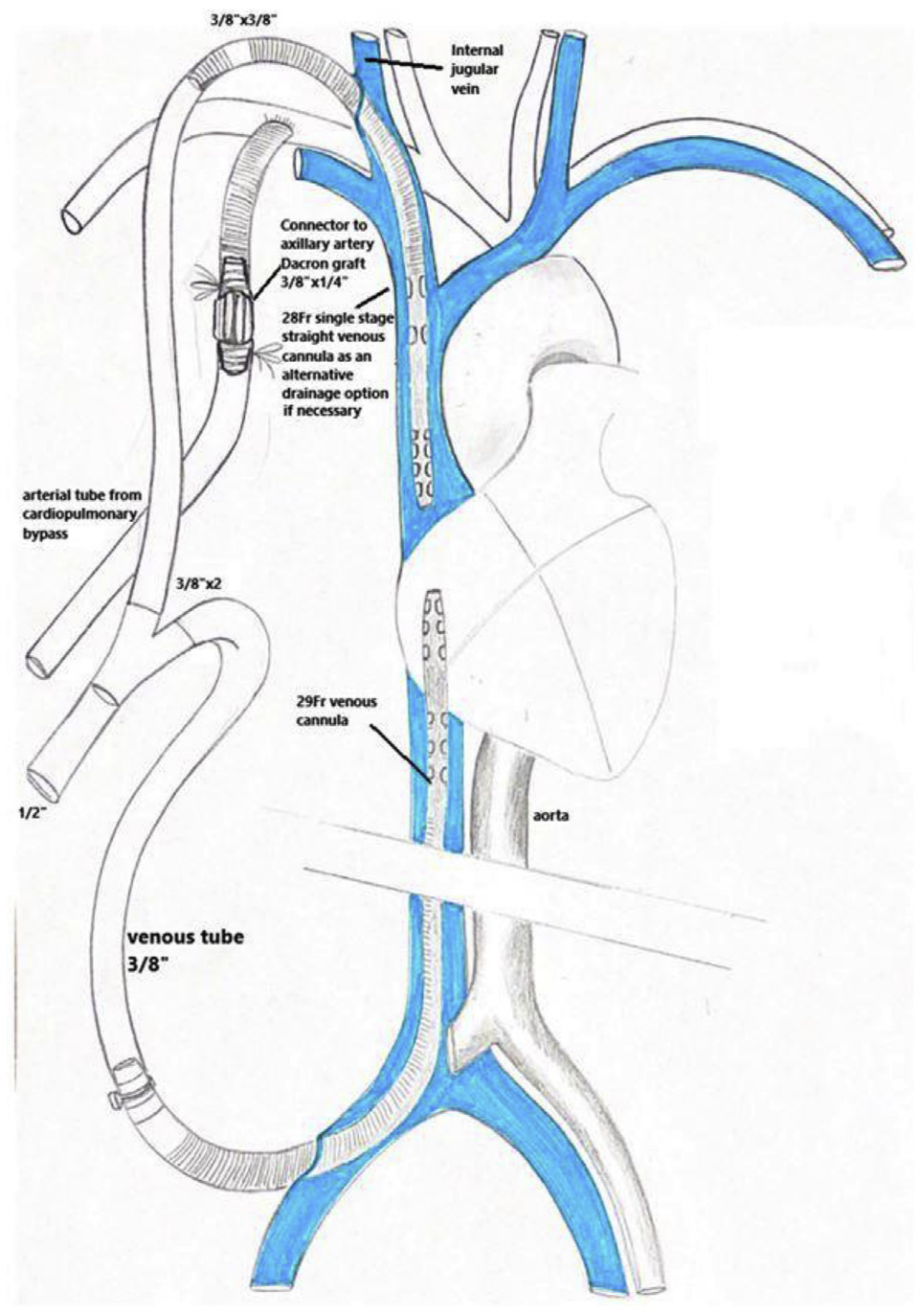
Cannulation strategy for connection to the cardiopulmonary bypass (CPB) machine. The initial plan was the use of a 29F venous cannula; however, a 27F cannula was used because the patient's cardiac output was only 3.8 L. The jugular vein cannula shown in the drawing is optional in the case of poor venous drainage, and the cannula was not used during the procedure. The arterial cannula was connected to the axillary artery via an 8-mm Dacron graft. A partial bypass strategy with an average flow of 3.5 L/min was obtained. Fluid was administered to maintain a target mean arterial pressure of 75 mm Hg, central venous pressure of 5 mm Hg, and cerebral mixed venous oxygen saturation of 45% to 50%.

Surgery was performed 33 days after the patient’s first symptoms and 17 days after admission. Aspirin, dalteparin, and atorvastatin had been administered since admission, with stable symptoms of slightly reduced cognition and left-sided neglect. General anesthesia was induced. Monitoring using near infrared spectroscopy (NIRS) was applied for cerebral oximetry monitoring (INVOS OEM solution for Mindray; Medtronic, Dublin, Ireland). Standard exposure of the right carotid artery was performed. The right axillary artery was accessed by cutdown and an 8-mm polyester graft was anastomosed end-to-side and connected to the CPB tubing. A 27F cannula was placed in the inferior vena cava through ultrasound-guided percutaneous access from the right femoral vein (Fig 3).

The CPB was started at an activated clotting time of 411seconds, and cooling of the patient was controlled by the machine using a partial bypass strategy. We aimed for a temperature just >30°C to avoid ventricular fibrillation. The mean arterial pressure was maintained at a target of 75 mm Hg. At 31°C, carotid artery clamping was performed and a longitudinal arteriotomy revealed an ulcerated heterogeneous plaque that was obstructing most of the proximal ICA. After occlusion, NIRS revealed an ipsilateral decrease from 61% to 22% and a contralateral decrease from 65% to 43%. An 8F, double-lumen, T-port carotid artery shunt was successfully placed, after which, the right NIRS value increased to 35%. The left remained just >40%. CEA was completed, and the arteriotomy was closed using a bovine pericardial patch. Flow was re-stablished to the ECA before the ICA. The NIRS values rapidly increased to 62% and 68% on the right and left sides, respectively. Measurement of the ICA flow showed a doubling compared with before CEA. After rewarming to 36°C, CPB was successfully discontinued. General anesthesia was reversed in the intensive care unit, and the patient awoke without new neurologic symptoms. She was moved to the bed ward on postoperative day 1 and transferred for rehabilitation on day 3. Acetylsalicylic acid as monotherapy was prescribed. After 1 month in her community hospital for stroke rehabilitation, her improvement was satisfactory, despite some remaining neurologic deficits. No new ischemic events had occurred by 5 months after the procedure. Her asymptomatic coronary disease was considered stable with conservative treatment. Simultaneous treatment appeared of low benefit,^8^ even with hypothermia,^9^ which supported this decision. After rehabilitation, she remained asymptomatic and refused coronary revascularization.

## Discussion

A dramatic bilateral NIRS decrease occurred during carotid clamping, and this remained low despite shunt application. Excessive manipulation of the narrow distal ICA was undesirable, and the slight NIRS increase after shunt placement was considered acceptable. The successful outcome suggests a correct indication and a protective effect of the induced hypothermia.

Transcarotid artery revascularization has been reported to be suitable for high-risk patients, including contralateral occlusion.^10^ Even if brief, flow reversal is a mandatory part of this procedure, and no description of the CoW or vertebral arteries was provided in the ROADSTER (safety and efficacy study for reverse flow used during carotid artery stenting procedure) studies. The procedure safety for the present patient is, therefore, uncertain.

CPB is a costly, time-consuming, and potentially harmful strategy; however, we still considered CPB to provide a treatment option for symptomatic patients with complex vascular pathology and a high risk of periprocedural hypoperfusion (Table).

**Table:** Suggested patient properties for consideration of carotid endarterectomy (CEA) under cardiopulmonary bypass (CPB)

All the following
Life expectancy >12 months
Symptomatic carotid stenosis
No contraindications for general anesthesia
High-degree ipsilateral ICA stenosis (>70%)
CEA anatomically favorable
No previous CEA or other neck surgery
No previous neck irradiation
ICA lesion below jaw angle
Anatomically suitable for peripheral cannulation
At least one of the following
High risk of perioperative hypoperfusion
Contralateral ICA occlusion
Precerebral multivessel disease
Malfunctioning CoW
No multivessel disease but anatomically unsuitable for transcarotid artery revascularization
Severe common carotid artery disease
Short common carotid artery (<5 cm)

*CoW,* Circle of Willis; *ICA,* internal carotid artery.

CEA has been ideally recommended within the first week after the index event.^11^ Our 33-day lag resulted from patient delay, a wrong initial primary care diagnosis of peripheral nerve injury, delayed transfer to the university hospital, and planning of the strategy and supplementary investigations. The crescendo tendency of ischemic events still suggests that even delayed carotid treatment would be appropriate.

## Conclusions

We believe that detailed vascular imaging studies, including of the CoW, is an important and possibly under-addressed evaluation for targeting the best individualized strategy. The results from the present case suggest that clinically induced hypothermia represents an option for high-risk patients.
